# The Orthostatic Hypotension Questionnaire in Swedish tested in patients with parkinsonism

**DOI:** 10.1002/brb3.1746

**Published:** 2020-07-07

**Authors:** Andreas Olsson, Mia Olsson, Artur Fedorowski, Peter Hagell, Klas Wictorin

**Affiliations:** ^1^ Division of Clinical Sciences Helsingborg Department of Clinical Sciences Lund Faculty of Medicine Lund University Lund Sweden; ^2^ Departments of Cardiology and Clinical Sciences Faculty of Medicine Lund University Malmö Sweden; ^3^ The PRO‐CARE Group Faculty of Health Sciences Kristianstad University Kristianstad Sweden

**Keywords:** orthostatic hypotension, Parkinson’s disease, rating scale, reliability, translation, validity

## Abstract

**Background:**

Orthostatic hypotension (OH) is common among older people and in particular in conditions like Parkinson’s disease (PD). The OH Questionnaire (OHQ) has been proposed as a useful patient‐reported assessment tool consisting of the OH Symptom Assessment (OHSA), OH Daily Activity Scale (OHDAS), and a composite score.

**Aims of the Study:**

To translate the OHQ into Swedish and assess its psychometric properties.

**Methods:**

Following forward–backward translation, the Swedish OHQ was field‐tested (*n* = 6) for relevance, comprehensibility, and respondent burden. It was then tested regarding scaling assumptions, targeting, reliability, and construct validity in persons with PD (*n* = 27) and multiple system atrophy (*n* = 2).

**Results:**

The Swedish OHQ was considered relevant and easy to use, with a mean completion time of 5.3 min. Scaling assumptions were acceptable for OHSA and OHDAS (corrected item‐total correlations, .30–.67) but not for the total score (.12–.69). Floor/ceiling effects were ≤3.4% and reliability was >.64. Construct validity was supported by expected correlations with the SCOPA‐AUT, RAND‐36, and blood pressure measurements.

**Conclusions:**

The Swedish OHQ was well received, and psychometric results suggest that the OHQ (particularly the OHDAS) is a useful tool for OH assessment in parkinsonian disorders. Further testing in larger samples is needed.

## 1 INTRODUCTION

The prevalence of orthostatic hypotension (OH) increases with age, and this condition may be detected in 10–30% of elderly persons (Low, 2008). OH is particularly common in Parkinson’s disease (PD), recently estimated to affect more than 65% of all patients within 7 years of diagnosis (Hiorth, Pedersen, Dalen, Tysnes, & Alves, 2019). The classical form of OH is defined as a sustained fall in systolic blood pressure (BP) of at least 20 mmHg or diastolic BP of at least 10 mmHg within 3 min of standing (Freeman et al., 2011). Since single or repeated orthostatic BP measurement cannot always reliably detect OH, or predict the occurrence or magnitude of OH‐related symptoms, Kaufmann, Malamut, Norcliffe‐Kaufmann, Rosa, and Freeman (2012) developed the Orthostatic Hypotension Questionnaire (OHQ) as a self‐reporting instrument to help fill this gap. The OHQ has been proposed as a valid tool in clinical research (Hauser, Biaggioni, Hewitt, & Vernino, 2018) and practice (Frith & Newton, 2016).

The OHQ (Table 1) is composed of two parts: the 6‐item OH Symptom Assessment (OHSA), assessing OH symptom severity, and the 4‐item OH Daily Activity Scale (OHDAS), assessing the impact of OH on daily activities. In addition, a composite 10‐item OHQ score has also been suggested (Kaufmann et al., 2012). All items are scored 0 through 10 (higher scores = worse) and summed into the respective total scores. It has been translated into multiple languages (H. Kaufmann, personal communication), but not yet into any Scandinavian language.

**Table 1:** Item and response category wording of the OHQ, English and Swedish versions

Orthostatic Hypotension Questionnaire Symptom assessment (OHSA) Graded on a Likert scale with a minimum of 0 (none) and a maximum of 10 (worst possible)	
English version	Swedish version
Dizziness, lightheadedness, feeling faint, or feeling like you might black out Problems with vision (blurring, seeing spots, tunnel vision, etc.) Weakness Fatigue Trouble concentrating Head/neck discomfort	Yrsel, svimningskänsla, upplevelse av att du kan komma att förlora medvetandet Problem med synen (suddighet, upplevelse av fläckar, tunnelseende, etc) Svaghet Trötthet Problem med koncentration Obehag i huvud/nacke
Orthostatic Hypotension Daily Activity Scale (OHDAS) Graded on a Likert scale with a minimum of 0 (no interference) and a maximum of 10 (complete interference). Including checkbox for each item: “Cannot do for other reasons”	
English version	Swedish version
Activities that require standing for a short time Activities that require standing for a long time Activities that require walking for a short time Activities that require walking for a long time	Aktiviteter som krävt att man står under en kort tid Aktiviteter som krävt att man står under en lång tid Aktiviteter som krävt att man går under en kort tid Aktiviteter som krävt att man går under lång tid

Note

The full, formatted scale (including respondent instructions) is available from the corresponding author.

Here, we translated the OHQ into Swedish, assessed the translation and tested its psychometric properties in persons with parkinsonian disorders.

## 2 METHODS

The study was approved by the local ethics committee and conducted in accordance with the declaration of Helsinki.

The translation was made using the forward–backward method by a panel of three experts in the fields of OH, PD, and patient‐reported outcome assessment. A field‐test regarding relevance, comprehensibility, and respondent burden (completion time) was performed with six persons with PD (three men) aged 64–85 years and diagnosed with PD since 4–7 years.

During the second half of 2018, a total of 41 persons with PD (*n* = 39) or multiple system atrophy (MSA) (*n* = 2), and with documented or suspected OH‐related symptoms, gave written consent and were screened for inclusion at the neurology outpatient clinics at three hospitals in southern Sweden. Patients were excluded either because they did not fulfill the OH consensus criteria (*n* = 7) or were found unable to manage the study protocol due to advanced cognitive or motor disability (*n* = 5). Twenty‐nine individuals (27 with PD and 2 with MSA) were included (Table 2). At a single visit for each patient, data were collected from patient records, orthostatic BP measurements (after 10 min of supine rest; directly upon standing and after 3 and 5 min of standing; using an automated device), clinical assessments using the Unified Parkinson’s Disease Rating Scale (UPDRS) part III (motor score) (Movement Disorder Society Task Force on Rating Scales for Parkinson's D, 2003), and the Montreal Cognitive Assessment (Nasreddine et al., 2005), and self‐report using the Swedish OHQ, the Scales for Outcome in Parkinson’s disease—Autonomic (SCOPA‐AUT) (Visser, Marinus, Stiggelbout, & Van Hilten, 2004), and the generic health status questionnaire RAND‐36 (Hays & Morales, 2001).

**Table 2:** Sample characteristics

Age (years), mean (*SD*)	72.0 (7.3)
Men/Women, *n* (%)	20 (69)/9 (31)
UPDRS III (score), median (q1–q3), mean (*SD*)	22 (16–28), 22 (11)
MoCA (score), mean (q1–q3)	24 (22–27)
PD duration/medication (years), mean (*SD*)	5.8 (4.5)
PD symptoms (years), mean (*SD*)	7.7 (4.8)
OH systolic BP fall (mmHg), mean (*SD*)	40 (25)
LEDD equivalent (mg), mean (*SD*)	757 (370)
OHQ completion time (minutes), mean (*SD*)	5.3 (2)

Note

q1–q3, first and third quartile; LEDD, Levodopa equivalent daily dose; *SD*, standard deviation.

Psychometric analyses were performed for the subscales as well as for the composite OHQ, according to Classical Test Theory (CTT) (Hobart & Cano, 2009; Ware & Gandek, 1998), using IBM SPSS 25. *Scaling assumptions* (i.e., the legitimacy of summing item scores into total scores) were analyzed by corrected item‐total correlations (CITC). *Targeting* (i.e., how well the individual scores accorded with scale coverage) was evaluated by the distribution of scale scores, floor and ceiling effects, and skewness. *Reliability (internal consistency)* was tested by Cronbach’s coefficient alpha, and score homogeneity was assessed by the average interitem correlation. *Construct validity* was tested by correlating (Spearman’s rho) OHQ scores with the various domain scores of the SCOPA‐AUT and RAND‐36, as well as with the maximum orthostatic drop in systolic BP (after 3 and 5 min of standing). It was hypothesized that OHQ scores would show the strongest correlations with scores on the cardiovascular domain (CV) of the SCOPA‐AUT and the physical functioning (PF) and role limitations/physical health (RP) scales of the RAND‐36. Correlations between OHQ scores and orthostatic drop in systolic BP were expected to be moderate, but stronger for OHSA than OHDAS scores. For additional interpretation criteria, see Table 3.

**Table 3:** Summary of results

	OHSA	OHDAS	OHQ
Scaling assumptions			
Corrected item‐total correlation (min‐max)^a^	0.30–0.52	0.60–0.67	0.12–0.69
Targeting			
Possible score range (midpoint)	0–60 (30)	0–40 (20)	0–100 (50)
Mean (*SD*) score^b^	23 (9.8)	17 (9.1)	40 (16.2)
Median (q1–q3) score^b^	22 (15.5–32.5)	17 (11.5–22)	38 (30.5–52.5)
Min‐max score^c^	7–43	0–35	10–78
Floor/Ceiling effects (%)^d^	0/0	3.4/0	0/0
Min‐max item floor/ceiling effects (%)^d^	10−28/0–7	7−14/0–7	7−28/0–7
Skewness^e^	0.35	0.02	0.43
Reliability			
Coefficient α^f^	0.64	0.81	0.79
Coefficient α when item deleted (min‐max)^g^	0.55–0.64	0.75–0.79	0.75–0.82
Construct validity^h^			
SCOPA‐AUT	0.22	0.16	0.26
Gastrointestinal	−0.07	−0.07	−0.09
Urinary	0.19	0.07	0.19
Cardiovascular (CV)	0.39	0.34	0.40
Thermoregulatory	0.27	0.19	0.30
Pupillomotor	0.03	−0.08	−0.02
RAND‐36^i^			
Physical functioning (PF)	−0.50	−0.46	−0.53
Role limitations/physical health (RP)	−0.70	−0.36	−0.64
Role limitations/emotional problems	−0.33	−0.27	−0.37
Energy/fatigue	−0.32	−0.08	−0.25
Emotional well‐being	−0.32	0.09	−0.12
Social functioning	−0.31	−0.03	−0.19
Pain	−0.10	−0.28	−0.28
General health	−0.41	−0.15	−0.32
Systolic BP difference max	0.40	0.33	0.38

OHDAS, Orthostatic hypotension Daily Activity Scale; OHQ, Orthostatic hypotension Questionnaire; OHSA, Orthostatic hypotension Symptom Assessment.

^a^ Should ideally be ≥0.3 to support summation of item scores and ≥0.4 to support unidimensionality.

^b^ Should be similar to score range midpoint.

^c^ Should cover most of the scale’s possible score range.

^d^ Should be <15%–20%.

^e^ Should be greater than −1 and less than 1.

^f^ Should be ≥70 and ideally ≥80.

^g^ Should not increase compared with alpha for the full scale

^h^ Spearman’s rho, ranges from −1 to 1, where 0 = no correlation.

^i^ Correlations are negative due to opposite scoring directions (OHQ—higher is worse, RAND‐36—higher is better).

## 3 RESULTS

The Swedish OHQ was considered relevant (*n* = 6) and easy to understand (*n* = 6); the average completion time was 7.2 (*SD*, 2.9; min‐max 4–13) minutes.

Psychometric data are summarized in Table 3. The CITCs were lower than in previous studies on the OHQ, but still within the acceptable range of 0.3 or greater (Hobart & Cano, 2009) for the OHDAS and OHSA. For the OHDAS, all CITCs were ≥0.6 and for the OHSA, item 6 (head/neck discomfort) had a CITC of 0.3 and items 1, 3, and 5 had values between 0.3 and 0.4. For the total OHQ score, all CITCs were >0.3 except for item 6 (0.12). Score homogeneities were 0.54 (OHDAS), 0.24 (OHSA), and 0.29 (total OHQ score).

Targeting analysis suggested that the participants were not constrained by the scale range. Average total scores were relatively close to the scale midpoints and not notably skewed. Floor and ceiling effects were absent for the OHSA as well as for the composite OHQ, and the OHDAS had a floor effect of 3.4%, still well within acceptable levels of <15%–20% (Hobart & Cano, 2009).

Reliability of the OHSA was suboptimal (0.64; 95% CI, 0.40–0.81), whereas it was acceptable for the OHDAS (0.81; 95% CI, 0.67–0.90) and the OHQ composite score (0.79; 95% CI, 0.63–0.89). For the OHSA, alpha remained identical when item 6 was deleted and when this item was deleted from the total OHQ score, alpha increased (0.82), suggesting problems with, for example, construct conceptualization or multidimensionality.

Analysis of construct validity relative to SCOPA‐AUT showed the strongest correlations with the CV domain for all OHQ scores. For RAND‐36, the strongest correlations were found with the PF and RP scores. The maximum systolic BP fall correlated stronger with OHSA (0.40) than with OHDAS (0.33).

## 4 DISCUSSION

The Swedish OHQ was well received by persons with parkinsonian disorders, and overall the psychometric analysis showed similar results to that of the English original (Frith & Newton, 2016; Kaufmann et al., 2012).

For scaling assumptions, it is recommended that individual items in any scale should have a CITC of ≥0.3, and preferably ≥0.4, to support the idea that the items represent the same phenomenon and to legitimatize summation of item scores into a total score (Hobart & Cano, 2009). Altogether, the Swedish OHQ met these criteria, although the OHSA and particularly item 6 (head/neck discomfort) displayed some problems that were also reflected in the total OHQ score. However, excluding this item would result in a scale not covering a symptom like “coat hanger pain,” which is known to frequently occur in OH. In the current study, we did not find support for the use of a total OHQ score. This is also reasonable from a clinical perspective, since symptoms and daily activities represent different aspects of health (World Health Organization, 2001).

Targeting was good as was score reliability (i.e., internal consistency), except for the OHSA. However, its 95% reliability CI overlapped the recommended standard, suggesting that additional samples are needed for firmer conclusions.

As hypothesized for the analyses of construct validity, the strongest correlations were found with the cardiovascular (CV) domain of the SCOPA‐AUT and the PF and RP scales of the RAND‐36. The strong correlations reported by Kaufmann et al. (2012) also for the energy/fatigue and social functioning domains of the RAND‐36 may be explained by their study cohort including a large proportion of patients with, for instance, neuropathy rather than neurodegenerative disease. The moderate to low correlations between the Swedish OHQ and single objective BP measurements further support the notion that a self‐reported questionnaire can be a complementary and even more valuable and sensitive tool for the detection of OH.

The present study was limited by its relatively small sample. However, interpretations of psychometric data according to CTT have been found to be stable with samples of about this size when compared to larger samples (Hobart, Cano, Warner, & Thompson, 2012). The cross‐sectional nature of the study prevented assessment of psychometric properties such as test–retest stability and responsiveness, and the use of more modern methodology such as Rasch measurement theory (Hobart & Cano, 2009). Since this study focused on people with PD and MSA, it is not clear whether or not our results are applicable to all patients with OH, regardless of etiology. However, based on the previous studies on the English version (Frith & Newton, 2016; Kaufmann et al., 2012), there is no reason to believe that the Swedish OHQ could not be reliably used in other patient groups. The study was also limited by the necessity to exclude some patients due to advanced disease. However, this was necessary because of inabilities to complete the self‐reports due to severe cognitive difficulties, or to maintain a standing position during the OH measurements.

In conclusion, the Swedish OHQ was well accepted by respondents and displayed promising psychometric results. As such, it appears to be a useful tool in assessing the burden of OH. However, our results, in this particular cohort of parkinsonian patients, suggest that the use of a composite OHQ can be problematic and that the OHSA could represent a symptom checklist rather than a scale. Again, differences in the psychometric results, compared with the studies of Kaufmann et al. (2012) and Frith and Newton (2016) on the English OHQ, could be due to their larger and more heterogeneous samples. Clearly, further and more extensive studies are needed for firmer testing and conclusions, also among patients with different underlying diseases and in the evaluation of OH treatment.

## CONFLICT OF INTEREST

No conflict of interest to report.

## AUTHOR CONTRIBUTIONS

AO, PH, and KW designed the study. AO and MO acquired the data. AO, AF, PH, and KW analyzed and interpreted the data. AO drafted the manuscript. AO, MO, AF, PH, and KW critically revised the manuscript. PH and KW supervised the study. All authors have read and approved of the final version.

### Peer Review

The peer review history for this article is available at https://publons.com/publon/10.1002/brb3.1746.

## Data Availability

The data that support the findings of this study are available from the corresponding author, K.W., upon request.
